# The albumin–bilirubin score as a predictor of outcomes in Japanese patients with PBC: an analysis using time-dependent ROC

**DOI:** 10.1038/s41598-020-74732-3

**Published:** 2020-10-20

**Authors:** Takanori Ito, Masatoshi Ishigami, Hikaru Morooka, Kenta Yamamoto, Norihiro Imai, Yoji Ishizu, Takashi Honda, Daisaku Nishimura, Toshifumi Tada, Satoshi Yasuda, Hidenori Toyoda, Takashi Kumada, Mitsuhiro Fujishiro

**Affiliations:** 1 Department of Gastroenterology and Hepatology, Nagoya University Graduate School of Medicine, 65 Tsurumai-cho, Showa-ku, Nagoya, 466-8550 Japan; 2 Department of Gastroenterology, Toyota Kosei Hospital, Toyota, Japan; 3 Department of Internal Medicine, Himeji Red Cross Hospital, Himeji, Japan; 4 Department of Gastroenterology and Hepatology, Ogaki Municipal Hospital, Gifu, Japan; 5 Department of Nursing, Gifu Kyoritsu University, Gifu, Japan

**Keywords:** Gastroenterology, Hepatology

## Abstract

The albumin–bilirubin (ALBI) score is calculated using only serum albumin and bilirubin levels, and was developed as a simple method to assess hepatic function. In this study, a total of 409 patients with primary biliary cholangitis (PBC) were enrolled between March 1990 and October 2018. The predictive performances of the ALBI score and other well-established prognostic scores were compared using time-dependent receiver operating characteristic (ROC) analysis. During the follow-up period, 60 patients died, 45 due to liver-related diseases and 15 due to non-liver-related diseases, and 16 patients underwent liver transplantation. Time-dependent ROC analysis showed that the ALBI score has higher the areas under the ROC curves (AUROCs) than the Child–Pugh (C–P) score at each time point; AUROCs at 3, 5, and 10 years after the start of follow-up were 0.94, 0.91, and 0.90 for the ALBI score, and 0.89, 0.88, and 0.82 for the C–P score, respectively. The ALBI score showed the highest AUROCs within 2 years after the start of observation; beyond 2 years, however, the Mayo score had better prognostic ability for mortality and liver transplantation. The ALBI score/grade, derived from objective blood tests, and the Mayo score were superior prognostic tools in PBC patients.

## Introduction

Primary biliary cholangitis (PBC) is an autoimmune chronic liver disease, which is characterized by destruction of the intrahepatic bile ducts, resulting in cholestasis and progressive fibrosis. The majority of PBC patients are asymptomatic at the time of diagnosis, and the clinical course is typically slow and progressive, but can differ significantly among individuals^1,2^. Although the overall 5-year survival rate of patients with PBC is 80–90%, a significant proportion of patients suffer from cirrhosis-related complications or hepatic malignancies^3,4^.

To distinguish patients at high risk of developing complications, several non-invasive prognostic markers have been used in patients with PBC, such as the Child–Pugh (C–P) score/classification, the model of end-stage liver disease (MELD), the Mayo risk score, and the Newcastle model^5–8^. All of these markers include the serum bilirubin level; in fact, the guidelines of the American Association for the Study of Liver Diseases (AASLD) state that serum bilirubin level is the most important factor for predicting prognosis in patients with PBC^1^.

The C–P classification system is one of the most commonly used methods worldwide for assessing hepatic function^9^. However, the C–P score/classification system includes subjective components, such as ascites and encephalopathy, and interrelated factors such as serum albumin and ascites. Recently, a simple new method was developed to assess hepatic function; known as the albumin–bilirubin (ALBI) score/grade, it is calculated using only serum albumin and bilirubin values^10^.

However, it is not yet clear whether the ALBI score/grade can serve as a novel biomarker capable of predicting prognosis in patients with PBC. Therefore, in this study, we evaluated the impact of several serum prognostic markers, including the ALBI score/grade, for predicting the prognosis of Japanese patients with PBC in the short and long term, using time-dependent receiver operating characteristic (ROC) analysis.

## Results

### Patient characteristics

The characteristics of the study patients are shown in Table 1. The mean age was 59.5 years, and there was a predominance of females (83.4%). The mean values of the ALBI score, C–P score, Mayo risk score, MELD score, and FIB-4 index were − 2.59, 5.9, 5.10, 8.06, and 2.26, respectively. The mean follow-up period was 8.8 years. Almost all patients (n = 379, 92.7%) were treated with ursodeoxycholic acid (UDCA; 600–900 mg/per day), and 41 patients (10.0%) who have already underwent the UDCA treatment at the start of follow-up. In addition, 122 (29.8%) patients underwent treatment with bezafibrate and 5 (1.2%) received corticosteroids. These five patients were treated with corticosteroids due to the comorbidities (rheumatoid arthritis, n = 3; Sjögren's syndrome, n = 1; and aplastic anemia, n = 1). One hundred eighty-five (45.2%) had the history of liver biopsy, and Scheuer’s stage was I for 138 cases (74.6%), II for 31 cases (16.8%), III for 14 cases (7.57%), and IV for 2 cases (1.1%). Only 19 patients (4.6%) with a history of HCC met the Milan Criteria or experienced a complete cure. During the follow-up period, 60 (14.7%) patients died, 45 due to liver-related disease and 15 due to non-liver related disease, and 16 (3.9%) patients underwent liver transplantation.

**Table 1:** Patient characteristics.

	(n = 409)
Age (years)	59.5 ± 12.7
Sex (female/male)	341 (83.4%)/68 (16.6%)
Platelet count (× 10^4^/m^3^)	21.3 ± 10.03
AST (U/L)	79 ± 166
ALT (U/L)	87 ± 223
γ-GTP (U/L)	87 ± 223
ALP (U/L)	609 ± 446
Total bilirubin (mg/dL)	1.39 ± 2.87
Albumin (g/dL)	3.92 ± 0.59
Prothrombin time (%)	99.0 ± 22.7
Immunoglobulin M (mg/dL)	436 ± 366
Anti-mitochondrial antibody positive (%)	373 (91.2%)
Child–Pugh classification (A/B/C)	321 (78.5%)/62 (15.2%)/26 (6.4%)
Child–Pugh score	5.9 ± 1.7
ALBI score	− 2.59 ± 0.65
FIB-4 index	2.26 ± 4.05
MELD score	8.06 ± 3.44
Mayo risk score	5.10 ± 2.00
UDCA/bezafibrate/steroid use	379 (92.7%)/122 (29.8%)/5 (1.2%)
History of liver biopsy (+ / −)	185 (45.2%)/224 (54.8%)
Scheuer’s stage (I/II/III/IV)	138 (74.6%)/31 (16.8%)/14 (7.57%) /2 (1.1%)
History of hepatocellular carcinoma (+ / −)	19 (4.6%)/390 (95.4%)
Event (death/liver transplantation)	60 (14.7%)/16 (3.9%)
Liver-related/non-liver-related deaths	45 (11.0%)/15 (3.7%)
Observation period (years)	8.8 ± 7.1

Continuous variables are expressed as mean ± SD (standard deviation).

*AST* aspartate aminotransferase; *ALT* alanine aminotransferase; *γ-GTP* γ-glutamyl transpeptidase; *ALP* alkaline phosphatase; *ALBI* albumin–bilirubin; *FIB-4* Fibrosis-4; *MELD* model of end-stage liver disease; *UDCA* ursodeoxycholic acid.

### Cumulative survival rate, incidence rates of death and liver transplantation, and causes of death

**Figure 1:**
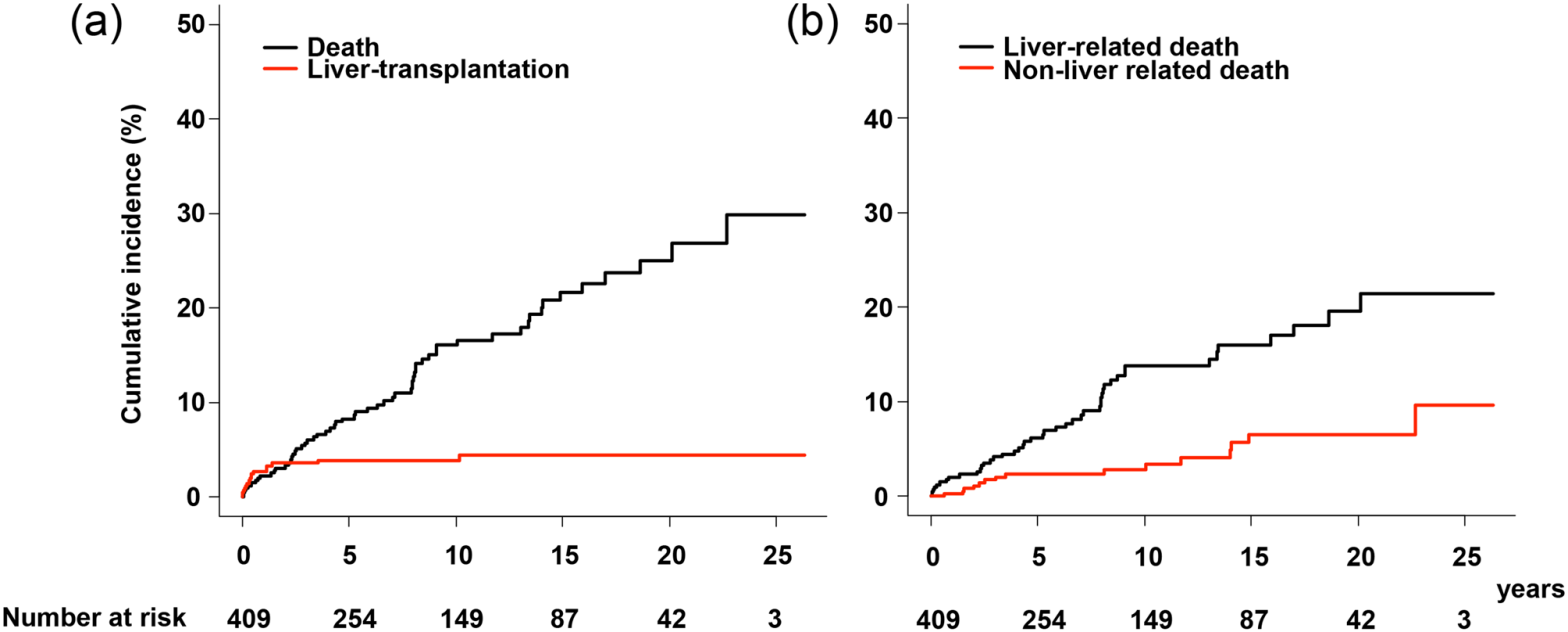
Incidence rates of liver-related/non-liver-related death and liver transplantation in all patients. (**a**) Cumulative incidence rates were 2.3%, 8.3%, 16.1%, 21.7%, and 25.0% for all-cause death, and 2.8%, 3.9%, 3.9%, 4.4%, and 4.4% for liver transplantation at 1, 5, 10, 15, and 20 years, respectively. (**b**) Cumulative incidence rates were 6.2%, 13.8%, 16.0%, and 19.5% for liver-related death, and 2.4%, 2.9%, 6.5%, and 6.5% for non-liver-related death at 5, 10, 15, and 20 years, respectively.

The overall and liver transplantation-free survival rates of patients at 5, 10, 15, and 20 years were 87.8% (95% confidence interval [CI], 83.9–90.8%), 80.0% (95% CI, 74.9–84.2%), 73.9% (95% CI, 67.6–79.2%), and 70.5% (95% CI, 63.2–76.6%), respectively (Supplementary Fig. S1 online). The 1-, 5-, 10-, 15-, and 20-year cumulative incidence rates of all-cause death were 2.3% (95% CI, 1.1–4.1%), 8.3% (95% CI, 5.7–11.5%), 16.1 (95% CI, 12.0–20.7%), 21.7 (95% CI, 16.4–27.4%), and 25.0% (95% CI, 18.8–31.8); those of liver transplantation were 2.8% (95% CI, 1.5–4.7%), 3.9% (95% CI, 2.3–6.2%), 3.9% (95% CI, 2.3–6.2%), 4.4% (95% CI, 2.6–7.0%), and 4.4% (95% CI, 2.6–7.0%) (Fig. 1a). The 5-, 10-, 15-, and 20-year cumulative incidence rates of liver-related death were 6.2% (95% CI, 3.9–9.1%), 13.8% (95% CI, 9.9–18.3%), 16.0% (95% CI, 11.5–21.1%), and 19.5% (95% CI, 13.8–26.0%); those of non-liver-related death were 2.4% (1.1–4.5%), 2.9% (95% CI, 1.4–5.2%), 6.5% (95% CI, 3.5–10.9%), and 6.5% (95% CI, 3.5–10.9%) (Fig. 1b). Of patients with liver-related deaths, 13 died due to HCC and 32 died due to liver cirrhosis-related complications other than HCC. In the remaining 17 patients with non-liver-related deaths, nine died due to extra-hepatic malignancies and eight died of other causes (Supplementary Table S2. online).

### Factors associated with prognosis by Fine-Grey proportional hazards models

Table 2 shows that the following parameters were selected as significant risk factors: age, C–P score, ALBI score, FIB-4 index, MELD score, and Mayo risk score for liver-related death and liver transplantation; age, male, C–P score, ALBI score, FIB-4 index, and Mayo risk score for non liver-related death. Univariate analysis with Fine-Grey proportion hazards model showed most of prognostic factors used in this study were selected as independent risk factors associated both liver-/non liver-related deaths in PBC.

**Table 2:** Factors associated with prognosis with Fine-Grey proportional hazards model.

Variables	Liver-related death or liver-transplantation		Non liver-related death	
	HR (95% CI)	*P* value	HR (95% CI)	*P* value
Age	1.065 (1.041–1.090)	< 0.001	1.124 (1.056–1.197)	< 0.001
Gender; female	1		1	
Male	1.019 (0.679–1.529)	0.930	1.940 (1.159–3.248)	0.012
Child–Pugh score	1.798 (1.580–2.047)	< 0.001	1.243 (1.014–1.524)	0.036
ALBI score	5.521 (3.858–7.900)	< 0.001	2.331 (1.387–3.917)	0.001
FIB-4 index	1.046 (1.015–1.078)	0.003	1.047 (1.014–1.081)	0.005
MELD score	1.199 (1.118–1.285)	< 0.001	1.083 (0.995–1.173)	0.051
Mayo risk score	1.748 (1.531–1.997)	< 0.001	1.350 (1.168–1.559)	< 0.001

*HR* hazard ratio; *CI* confidence interval; *ALBI* albumin–bilirubin; *FIB-4* Fibrosis-4; *MELD* model of end-stage liver disease.

### Time-dependent ROC analysis for overall survival and the incidence of liver transplantation

**Figure 2:**
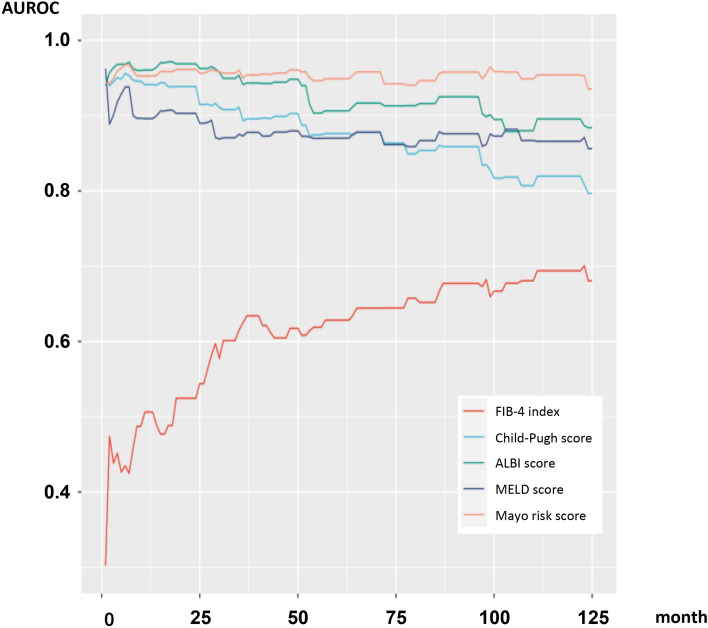
Time-dependent AUROCs for overall and liver transplantation-free survival after the start of follow-up. The albumin–bilirubin (ALBI) score demonstrated higher area under the receiver operating characteristic curves (AUROCs) for survival and liver transplantation than the Child–Pugh score at each time point. The Mayo risk score kept consistently higher AUROCs for outcome during the follow-up period; however, the ALBI score showed higher AUROCs than the Mayo risk score within 2 years after the start of observation. The AUROC of the Fibrosis-4 index was the lowest of all the markers. *AUROC* area under the receiver operating characteristic curve; *ALBI* albumin–bilirubin; *FIB-4* Fibrosis-4; *MELD* model of end-stage liver disease.

Figure 2 shows the plots of AUROCs for the ALBI score, C–P score, FIB-4 index, Mayo score, and MELD score for patient overall and liver transplantation-free survival from 1 to 10 years after the start of follow-up. The detailed ROC curves for each marker after the start of follow-up, obtained using time-dependent ROC analysis, are shown in Supplementary Fig. S3 online. Time-dependent ROC analysis showed that the predictive power of the ALBI score for overall mortality and the incidence of liver transplantation was superior to that of the C–P score for all years; the AUROCs at 3, 5, 7, and 10 years after the start of follow-up were 0.941, 0.906, 0.916, and 0.896 for the ALBI score, and 0.893, 0.876, 0.854, and 0.82 for the C–P score, respectively. Over the course of the follow-up period, the Mayo risk score gradually became superior to the other markers, with the highest AUROCs for mortality and liver transplantation; however, the value of AUROCs in ALBI score showed larger than the Mayo risk score within 2 years after the start of observation. The AUROCs of the FIB-4 index were the lowest of all the markers.

### Overall survival and liver transplantation-free rates based on the ALBI grade and C–P class

**Figure 3:**
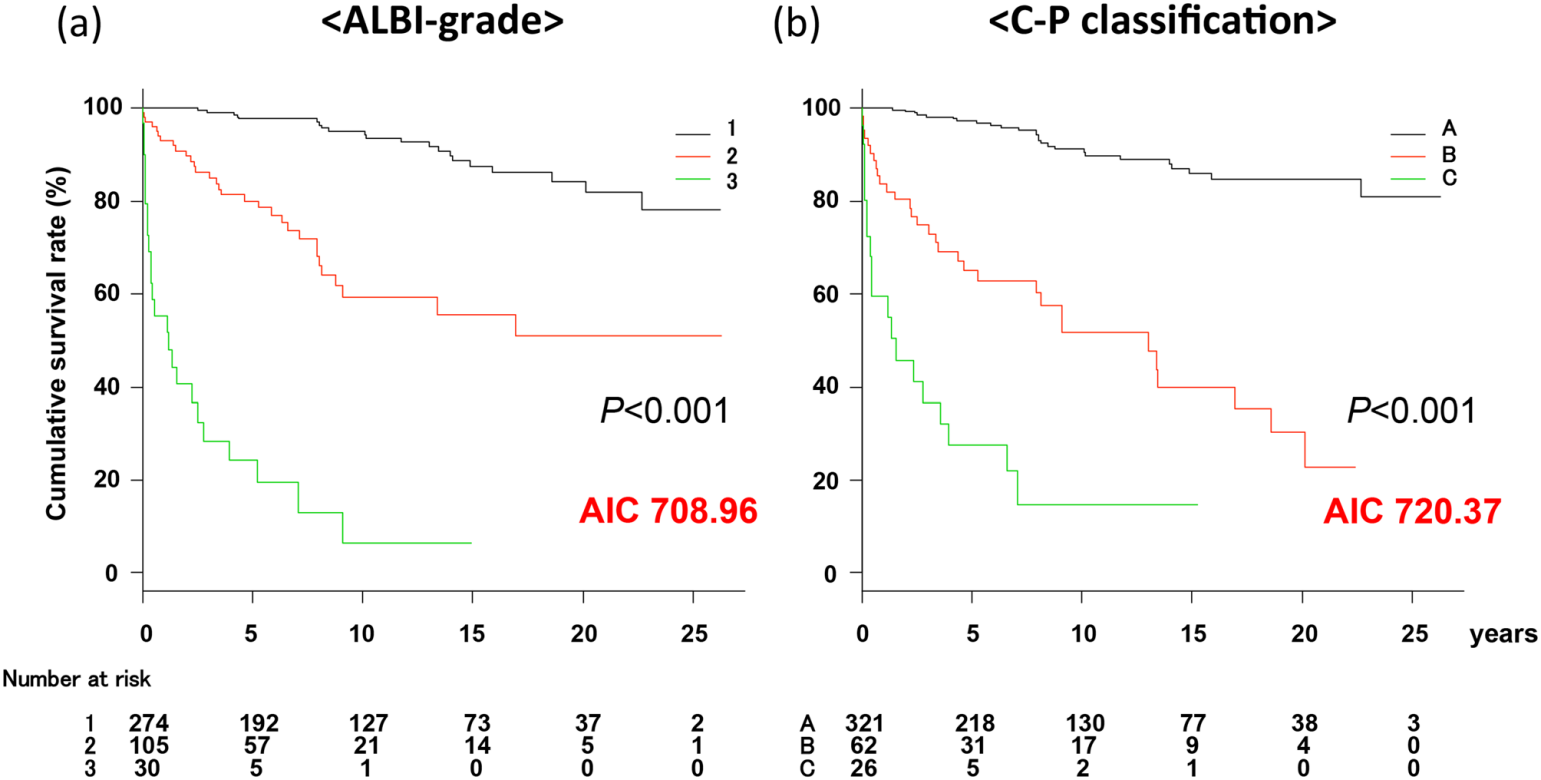
Overall survival and transplantation-free rates based on ALBI grade and Child–Pugh classification of PBC. Both the albumin–bilirubin (ALBI) grade and Child–Pugh (C–P) classification predicted patient outcomes with good discriminative ability. However, the Akaike’s information criterion (AIC) for the ALBI grade was better than that for the C–P classification (708.96 vs. 720.37). *ALBI* albumin–bilirubin; *C–P* Child–Pugh; *AIC* Akaike’s information criterion.

In patients classified as ALBI-grade 1 (n = 274), 2 (n = 105), and 3 (n = 30), the survival and liver transplantation-free rates were 97.7% (95% CI, 94.4–99.0%), 80.0% (95% CI 70.0–87.0%), and 24.3% (95% CI, 10.2–41.6%) at 5 years, 95.1% (95% CI, 90.6–97.4%), 59.3% (95% CI, 45.8–70.5%), and 6.5% (95% CI, 0.5–24.2%) at 10 years, and 87.5% (95% CI, 80.1–92.2%), 55.6% (95% CI, 41.0–67.9%), and 6.5% (95% CI, 0.5–24.2%) at 15 years, respectively (Fig. 3a). In addition, in patients categorized as C–P class A (n = 321), B (n = 62), and C (n = 26), the survival and liver transplantation-free rates were 97.3% (95% CI, 94.4–98.7%), 65.0% (95% CI, 51.0–75.9%), and 27.5% (95% CI, 11.4–46.4%) at 5 years, 91.2% (95% CI, 86.2–94.4%), 51.7% (95% CI, 36.5–65.0%), and 14.6% (95% CI, 3.1–34.5%) at 10 years, and 85.9% (95% CI, 79.1–90.6%), 39.8% (95% CI, 23.8–55.4%), and 14.6% (95% CI, 3.1–34.5%) at 15 years, respectively (Fig. 3b). Both the ALBI grade and C–P classification showed good discriminatory ability for prognosis (mortality and liver transplantation). However, the AIC of the ALBI grade was better than that of the C–P classification (708.96 vs. 720.37, respectively).

## Discussion

Although several prognostic scores have been reported to be associated with prognosis in PBC patients^11^, it was unclear whether these markers reflect prognosis in both the short and long term. Therefore, we compared these well-established predictive markers, including the ALBI score, using time-dependent ROC curves in PBC patients. In our cohort, although more than 70% of deaths in patients with PBC were due to liver-related disease, time-dependent ROC analysis showed that the ALBI score and Mayo score had higher AUROCs than the other markers for predicting overall survival and the incidence of liver transplantation.

ROC analysis is commonly used to evaluate the discriminatory power of a continuous variable for a binary disease outcome. However, it is impossible to compare the prognosis determined using general ROC analysis because outcomes are time dependent. In addition, the Kaplan–Meier method, which is frequently used to investigate prognosis, requires optimal cutoff values for each marker. For instance, there is no established cutoff value for the Mayo score, and it is unclear whether the range of FIB-4 index cutoff values from 1.45 to 3.25, as determined in a previous study on hepatitis C virus infection^12^, is applicable to PBC. On the other hand, time-dependent ROC curves have been introduced for assessing the predictive power of diagnostic markers for time-dependent disease outcomes^13,14^. This analysis can compare multiple markers at the same time without the need to determine cutoff values. This is the first report in which the prognosis of PBC patients was assessed using continuous predictive markers and time-dependent ROC analysis.

This study clearly demonstrated that both the ALBI score and Mayo score, assessed at the start of follow-up, were strongly associated with outcomes (death or liver transplantation). Clinically, the C–P score/classification is commonly used to determine the prognosis of patients with chronic liver diseases or liver cancers^9,15,16^. However, the C–P score is limited by its use of arbitrary parameter cutoff values and by the fact that all five parameters are weighted equally, including two subjective parameters: the presence of ascites and the degree of hepatic encephalopathy. By contrast, the ALBI score is calculated using only serum albumin and bilirubin levels, both of which are incorporated in the conventional C–P score, and it does not require evaluation of prothrombin time, another parameter in the C–P classification system. Recently, the ALBI score/grade has been widely used for predicting outcomes, especially in the field of HCC^17–19^. Regarding the analysis of clinical outcomes in PBC, Fujita et al. and Chan et al. reported the utility of the ALBI score as a predictive marker^20,21^. However, both studies used the Kaplan–Meier method and/or Cox-proportional hazard model for outcome analysis, but neither statistical technique can adequately analyze both short- and long-term outcomes. Since most prognostic markers for PBC incorporate serum bilirubin and albumin levels, multivariate analysis is unsuitable for comparing these markers because these levels can be confounding factors. A strength of our study is that it evaluates outcome data using time-dependent ROC analysis comparing these studies at the same time points.

On the other hand, almost all prognostic markers used in the present study were selected as significant factors for predicting not only liver-related but also non-liver related events by univariate analysis using Fine and Gray proportional hazards models. In 15 cases died by non liver-related deaths, more than half patients (n = 8) died due to non-malignant diseases: acute pneumonia; n = 4, gastrointestinal bleeding other than varices bleeding; n = 3, acute pancreatitis; n = 1 (Supplementary Table S2. online). Especially, the incidence of pneumonia and GI bleeding were related to the progression of PBC, and prognostic markers for PBC might be able to affect HRs for these incidence.

Time-dependent ROC analysis showed that the AUROCs of ALBI score were higher than those of the Mayo score within 2 years after the start of observation. Conversely, the Mayo score had a high AUROC throughout the follow-up period. The Mayo score is one of the most widely accepted non-invasive markers for estimating the prognosis of patients with PBC^8,22^. The high, stable AUROC of the Mayo score indicated its efficacy in predicting both short- and long-term outcomes. However, one of its drawbacks is that like the C–P score, its calculation requires the presence of edema, a subjective parameter. Therefore, the ALBI score/grade has advantages in terms of simplicity and objectivity. Interestingly, the AUROC of the FIB-4 index, a non-invasive fibrosis marker used widely in clinical settings, was the lowest of all the markers, although it gradually increased over time. To reveal which factors in the component of FIB-4 index can be responsible for this change, we performed time-dependent ROC analysis using each single marker (Supplementary Fig. S4. online). The FIB-4 index is calculated with age, AST, ALT, and platelet counts, but this analysis revealed the age factor showed similar changes of the AUROCs in FIB-4 index, indicating that this shift seems to be strongly influenced by age factor. These results indicate that the FIB-4 index is not ideal for predicting prognosis in a short term. On the other hand, the AUROCs of total bilirubin were the highest, and those of albumin were lowest among these single markers. Therefore, the high AUROCs in ALBI score can be mainly affected by the bilirubin factor. However, the ALBI score that calculated by bilirubin and albumin showed higher AUROCs than bilirubin alone, suggesting that it could be used as a better prognostic marker by combining them. Previous reports showed that non-invasive fibrosis markers are essential for estimating outcomes in PBC^23,24^. The ALBI and Mayo scores, which include bilirubin, are superior to the FIB-4 index for predicting outcomes, including mortality and liver transplant-free survival, throughout the clinical course of patients with PBC.

This study has several limitations. First, this was a retrospective, hospital-based cohort study, which increases the risk of selection bias. We included patients with PBC from three clinical hospitals, but liver transplantation was performed only in Nagoya University Hospital. Since accessibility to liver transplantation was limited in the other two participated hospitals, there was a concern for analyzing outcomes. However, the ALBI score/grade showed identical prognostic results between these hospitals (data not shown) implying the accessibility did not affect the prognosis in PBC patients in our clinical settings. Second, although recent studies focusing on transplant-free survival after 1 year of UDCA therapy demonstrated the utility of two additional prognostic scores, the GLOBE score and UK-PBC score^25,26^, we were unable to assess prognosis using these scores. In this study, almost all patients (92.3%) underwent treatment with UDCA during follow-up, but we did not assess the response to UDCA. Our study included cases with rapid progression of liver dysfunction, and we investigated not only long-term but also short-term prognosis, i.e., within 1 year. However, the GLOBE and UK-PBC scores require data obtained at least 1 year after the start of UDCA therapy. In addition, 41 patients (10.0%) who have already started the UDCA treatment at the start of follow-up. To assess several additional markers, including the GLOBE and UK-PBC scores, further studies should recruit additional UDCA naïve patients with observation periods over 1 year. Third, histological confirmations of liver fibrosis were obtained in only some patients. In addition, in many cases there was a time lag from the start of follow-up to liver biopsy. Therefore, in this study we did not assess the correlation between outcome and pathological liver fibrosis. However, the strengths of our study included its long-term follow-up period and large number of patients.

In conclusion, our study showed that the ALBI score and Mayo score had high prognostic ability for predicting outcomes in PBC patients. An advantage of the ALBI score/grade is that it can be simply calculated by objective blood tests without subjective factors or invasive procedures. Therefore, the ALBI score/grade may be useful to determine treatment strategies for PBC patients. Conversely, the FIB-4 index has limited ability to predict prognosis in the short term. Clinicians following patients with PBC should be familiar with the characteristics of each of these predictive markers.

## Methods

### Patients

Between March 1990 and October 2018, a total of 435 patients were diagnosed with PBC at Nagoya University Hospital, Ogaki Municipal Hospital, and Toyota Kosei Hospital. The diagnosis of PBC was based on criteria by the Japan Society of Hepatology^16^, as follows: Patients with one of the following criteria should be diagnosed with PBC; (1) histologically confirmed chronic non-suppurative destructive cholangitis (CNSDC) with laboratory findings compatible with PBC; (2) positivity for anti-mitochondrial antibodies (AMAs) with histological findings compatible with PBC but in the absence of characteristic histological findings of CNSDC; and (3) no histological findings available, but positivity for AMAs as well as clinical findings and a course indicative of typical cholestatic PBC. The pathological stage of PBC was evaluated by an experienced pathologist who specialized in liver pathology based on the Scheuer’s classification as follows: stage 1, florid duct lesion; stage 2, ductular proliferation; stage 3, scarring; and stage 4, cirrhosis^27^.

Twenty-six patients were excluded for the following reasons: (1) insufficient follow-up or incomplete data, n = 2; (2) PBC-AIH (autoimmune hepatitis) overlap syndrome, n = 15; (3) suspected liver injury induced by concomitant nonalcoholic steatohepatitis from hepatic pathology, n = 1; (4) concomitant hepatitis B and/or C virus infection, n = 3; (5) hepatocellular carcinoma (HCC) that did not meet the Milan Criteria^28^, n = 5. Ultimately, 409 patients were enrolled in this retrospective study. We defined probable PBC-AIH overlap syndrome based on the combination of immunoglobulin G levels more than twice the upper limit of normal (ULN) and aminotransferase levels more than five times the ULN^29^. If we suspected overlap syndrome, the final diagnosis was performed based on pathological findings on liver biopsy^30^. Additionally, we excluded the patients who could improve their liver-related laboratory data by the administration of corticosteroids during the follow-up period as PBC-AIH overlap syndrome. In this study, for assessing liver transplantation-free survival in patients who met the Milan Criteria, we included patients with HCC who met the Milan Criteria at the time of diagnosis of PBC, as well as those with a history of complete cure of HCC. Regular surveillance was performed every 3–6 months using ultrasonography and/or blood tests, and included measurement of the tumor marker alpha-fetoprotein. Decisions regarding the treatment of each patient were based on Japanese treatment guidelines for PBC^16^. The study protocol was approved by the institutional review board of Nagoya University Hospital (No. 2019-0055) and was in compliance with the Helsinki Declaration. Informed consent in the present study was obtained in the form of opt-out on the website of Nagoya University Hospital, Ogaki Municipal Hospital, and Toyota Kosei Hospital.

### Calculation of prognostic parameters

The formulas of the MELD score, Mayo risk score for PBC, Fibrosis-4 (FIB-4) index, and ALBI score/grade were as follows:

- MELD score = (0.957 * ln(serum creatinine) + 0.378 * ln(serum bilirubin) + 1.120 * ln(INR) + 0.643) * 10; if on hemodialysis: calculate as creatinine 4.0 mg/d^5^.
- Mayo risk score = 0.051(age) + 1.209 log(bilirubin) − 3.304 log(albumin) + 2.754 log(prothrombin time sec) + 0.675(edema); edema: 0 = no edema without diuretics, 0.5 = edema without diuretic therapy or edema resolved with diuretic therapy, 1 = edema despite diuretic therapy^8^.
- FIB-4 index = aspartate aminotransferase (AST) [IU/L] * age [years]/platelet count [10^9^/L] * alanine aminotransferase (ALT) [IU/L]^1/2^^12^.
- ALBI score/grade = {log10 bilirubin (μmol/L) * 0.66} + {albumin (g/L) * − 0.085}. Grade 1 liver function corresponds to an ALBI score ≤  − 2.60, grade 2 corresponds to an ALBI score from − 2.60 to − 1.39, and grade 3 corresponds to an ALBI score >  − 1.39^10^.

In this study, we used the modified C–P score for PBC based on a previous report^16^. These prognostic markers were calculated at the start of follow-up at each institution and used for analysis of the prognosis in the present study.

### Statistical analysis

Continuous variables are expressed as mean ± SD (standard deviation). Categorical variables are expressed as number (percentage). Actuarial analysis of cumulative survival was performed using the Kaplan–Meier method based on C–P and ALBI grades, and differences were tested using the log-rank test. Discriminatory abilities of the scoring models were assessed using Akaike’s information criterion (AIC). Fine-Grey proportional hazards models were used to calculate hazard ratios (HRs) for overall disease-related and liver transplantation-related mortality. Statistical significance was defined as *P* < 0.05. Statistical analyses other than time-dependent ROC were performed with EZR (Saitama Medical Center, Jichi Medical University, Saitama, Japan), a graphical user interface for R (The R Foundation for Statistical Computing, Vienna, Austria)^31^. More precisely, it is a modified version of the R commander designed to add statistical functions frequently used in biostatistics. We used the “survivalROC” package, written for R, to assess marker performance using time-dependent ROC curves.

## Supplementary information

Supplementary Information

## Abbreviations

ALBI: Albumin–bilirubin
PBC: Primary biliary cholangitis
ROC: Receiver operating characteristic
AUROC: Area under the receiver operating characteristic curve
C–P: Child–Pugh
MELD: Model of end-stage liver disease
AASLD: American Association for the Study of Liver Diseases
AIH: Autoimmune hepatitis
HCC: Hepatocellular carcinoma
ULN: Upper limit of normal
AST: Aspartate aminotransferase
ALT: Alanine aminotransferase
γ-GTP: γ-Glutamyl transpeptidase
ALP: Alkaline phosphatase
SD: Standard deviation
AIC: Akaike’s information criterion
HR: Hazard ratio
CI: Confidence interval
UDCA: Ursodeoxycholic acid
CNSDC: Chronic non-suppurative destructive cholangitis
AMA: Anti-mitochondrial antibodies
