# Supplementary material for: The albumin–bilirubin score as a predictor of outcomes in Japanese patients with PBC: an analysis using time-dependent ROC
Source: Sci Rep. 2020 Oct 20;10:17812. doi: 10.1038/s41598-020-74732-3 (PMC7576583; doi:10.1038/s41598-020-74732-3)

(Supplemental information)

The albumin–bilirubin score as a predictor of outcomes in Japanese patients with PBC: An analysis using time-dependent ROC

Takanori Ito[1], Masatoshi Ishigami[1], Hikaru Morooka[1], Kenta Yamamoto[1], Norihiro Imai[1], Yoji Ishizu[1], Takashi Honda[1], Daisaku Nishimura[2], Toshifumi Tada[3], Satoshi Yasuda[4], Hidenori Toyoda[4], Takashi Kumada[5], Mitsuhiro Fujishiro[1]

1. Department of Gastroenterology and Hepatology, Nagoya University Graduate School of Medicine, Nagoya, Japan
2. Department of Gastroenterology, Toyota Kosei Hospital, Toyota, Japan
3. Department of Internal medicine, Himeji Red Cross Hospital, Himeji, Japan.
4. Department of Gastroenterology and Hepatology, Ogaki Municipal Hospital, Gifu, Japan
5. Department of Nursing, Gifu Kyoritsu University, Gifu, Japan.

Corresponding author: Takanori Ito, MD, PhD
Department of Gastroenterology and Hepatology, Nagoya University Graduate School of Medicine, 65 Tsurumai-cho, Showa-ku, Nagoya, 466-8550, Japan
TEL: +81-52-744-2169 / FAX: +81-52-744-2178
E-Mail: tahkun56@gmail.com

Supplementary Fig. S1

Overall survival rates in all patients

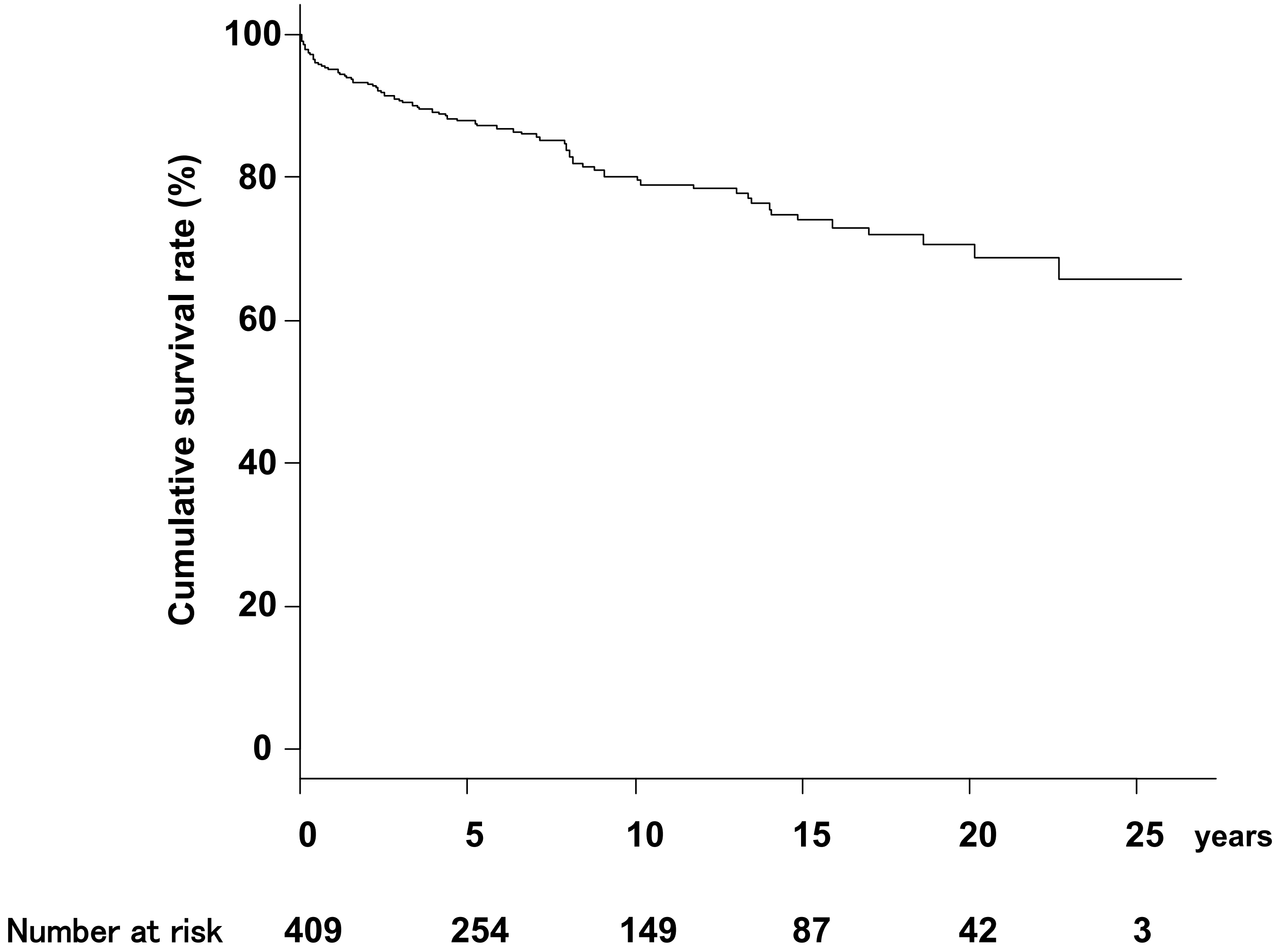

The respective 5, 10, 15, and 20-year cumulative overall and liver transplantation–free survival in all patients were 87.8%, 80.0%, 73.9%, and 70.5%.

Supplementary Table S2.
Causes of liver-/non liver-related deaths

Liver-related diseases	(N=45)	
	Hepatocellular carcinoma	13
	Liver-cirrhosis related complication	32
Non liver-related diseases	**(N=15)**	
Malignancies		
	Colorectal Cancer	2
	Gastric Cancer	1
	Pancreatic cancer	1
	Lung cancer	1
	Breast Cancer	1
	Malignant lymphoma	1
Total		7
Non malignant diseases		
	Acute pneumonia	4
	Gastrointestinal bleeding †	3
	Acute pancreatitis	1
Total		8

N, number. † This category did not include the bleeding from varices.

Supplementary Fig. S3

Time-dependent ROC curves of all markers for overall survival and the incidence of liver transplantation.

<FIB-4 index>

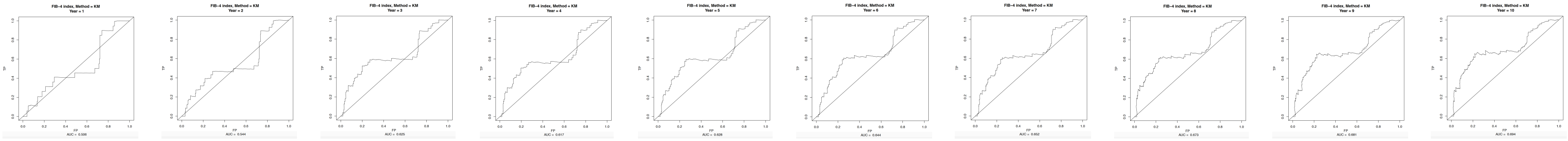

<Child-Pugh score>

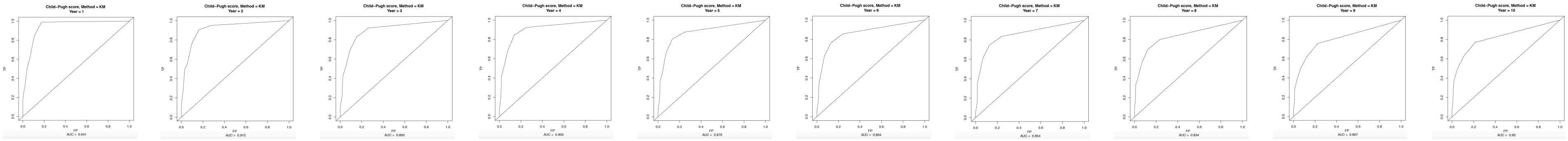

<ALBI score>

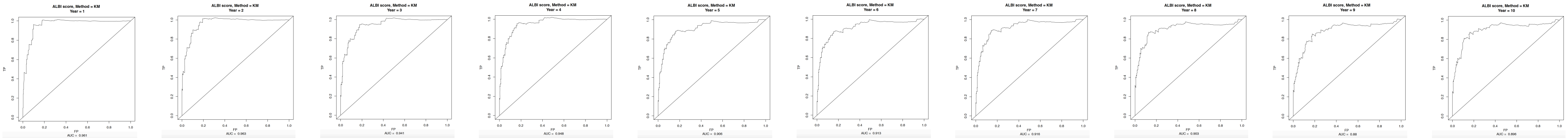

<MELD score>

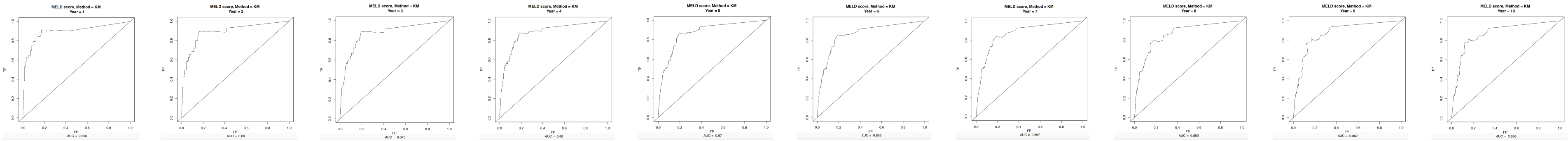

<Mayo risk score>

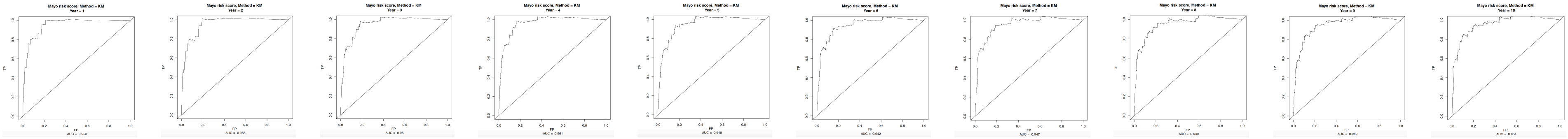

1-year 2-year 3-year 4-year 5-year 6-year 7-year 8-year 9-year 10-year

Supplementary Fig. S4

Time-dependent AUROCs for overall and liver transplantation-free survival with each parameter

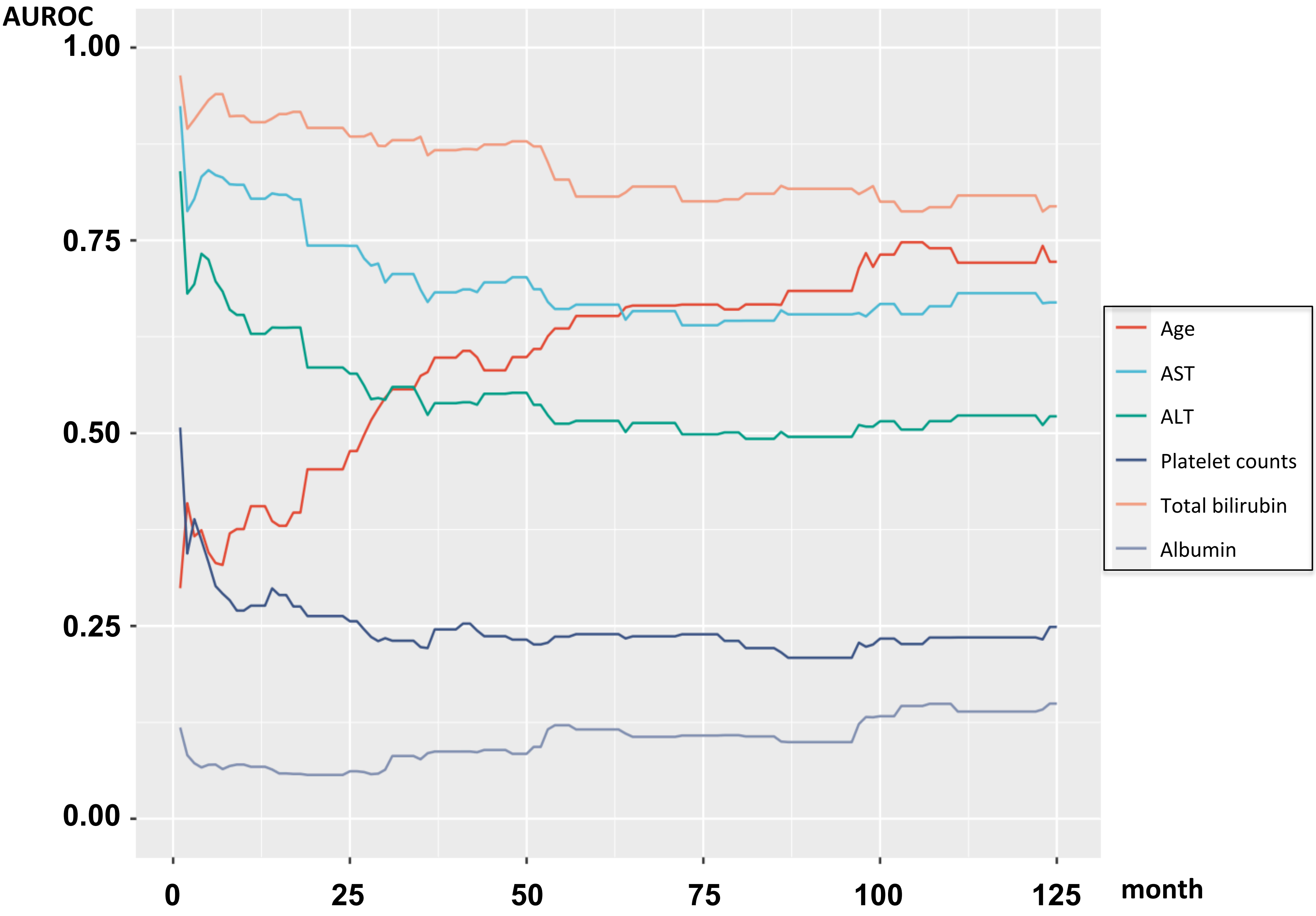

Supplement: Supplementary file 1 — Supplementary Information [file 41598_2020_74732_MOESM1_ESM.pdf]
